# Whisker array functional representation in rat barrel cortex: transcendence of one-to-one topography and its underlying mechanism

**DOI:** 10.3389/fncir.2012.00093

**Published:** 2012-11-27

**Authors:** Cynthia H. Chen-Bee, Yi Zhou, Nathan S. Jacobs, Beatrice Lim, Ron D. Frostig

**Affiliations:** ^1^Department of Neurobiology and Behavior, University of CaliforniaIrvine, CA, USA; ^2^Department of Biomedical Engineering, University of CaliforniaIrvine, CA, USA; ^3^Center for the Neurobiology of Learning and Memory, University of CaliforniaIrvine, CA, USA

**Keywords:** vibrissa, whisker, topography, column, intrinsic signal optical imaging, multi-unit, local field potential, lidocaine

## Abstract

The one-to-one relationship between whiskers, barrels, and barrel columns described for rat barrel cortex demonstrates that the organization of cortical function adheres to topographical and columnar principles. Supporting evidence is typically based on a single or few whiskers being stimulated, although behaving rats rely on the use of all their whiskers. Less is known about the cortical response when many whiskers are stimulated. Here, we use intrinsic signal optical imaging and supra- and sub-threshold electrophysiology recordings to map and characterize the cortical response to an array of all large whiskers. The cortical response was found to possess a single peak located centrally within a large activation spread, thereby no longer conveying information about the individual identities of the stimulated whiskers (e.g., many local peaks). Using modeling and pharmacological manipulations, we determined that this single central peak, plus other salient properties, can be predicted by and depends on large cortical activation spreads evoked by individual whisker stimulation. Compared to single whisker stimulation, the peak magnitude was comparable in strength and the response area was 2.6-fold larger, with both exhibiting a reduction in variability that was particularly pronounced (3.8x) for the peak magnitude. Findings extended to a different collection (subset) of whiskers. Our results indicate the rat barrel cortex response to multi-site stimulation transcends one-to-one topography to culminate in a large activation spread with a single central peak, and offer a potential neurobiological mechanism for the psychophysical phenomenon of multi-site stimulation being perceived as though a single, central site has been stimulated.

## Introduction

**Figure 1:**
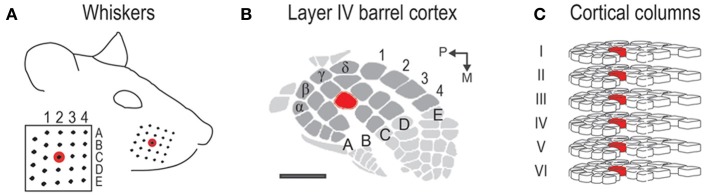
**Rat whisker-to-barrel system. (A)** The 24 largest whiskers located in rows A–E and arcs 1–4 plus the 4 Greek whiskers. Red = central whisker C2. **(B)** Barrel cortex subregion of layer IV primary somatosensory cortex. Anatomical representations (barrels) for the 24 whiskers are shaded in dark gray; C2 barrel shaded in red. Scale bar = 1 mm. **(C)** Cortical columns for the 24 whiskers. Each column contains neurons responding preferentially to a particular whisker. C2 column shaded in red.

The rat barrel cortex subdivision of the primary somatosensory system (for review see Fox, 2008) exquisitely demonstrates two fundamental principles of cortical functional organization. Each large whisker found on the snout (Figure 1A) is individually represented anatomically in layer IV barrel cortex in a topographical manner (Figure 1B), which adheres to the topographical principle of cortical organization. Each whisker is also individually represented functionally in a columnar manner in which neurons above, below, and within a barrel respond preferentially to the same whisker (Figure 1C). Thus, barrel cortex also adheres to columnar principles of cortical organization. For barrel cortex, note both principles of organization strongly convey a one-to-one mapping of the whiskers onto the cortex. What is known about the function of barrel cortex is largely based on stimulating a single or few whiskers. Less is known about the barrel cortex response when an entire whisker array (>20 + whiskers) is stimulated. Such characterization should be of interest as rats rely on all their whiskers (vibrissae), typically “whisking” them together (repetitive back-and-forth movements in the rostral-caudal axis at ~5–10 Hz rate) during tactile exploration (Carvell and Simons, 1990). In other words, rats are routinely subjected to stimulation of many whiskers rather than just one or few. As remarked upon by Petersen et al. (2009), the relevant parameter space for the ability of whiskers to influence each other's cortical response is rather large. Therefore, the cortex's response to the entire whisker array is likely not a simple extrapolation of previous findings based on stimulating two or several whiskers.

Thus far, the cortical response to whisker array stimulation has been explicitly investigated in only a couple of studies. Single unit response preference to a particular direction of global motion across the whisker array (Jacob et al., 2008) or spatiotemporal patterns of evoked potentials as a metal wire swept sequentially across the whisker array (Benison et al., 2006) have been characterized. To date, the total cortical activation spread responsive to whisker array stimulation [referred to as multi-whisker functional representation (MWFR)] has yet to be mapped and characterized in detail. In the present study, we studied MWFRs using intrinsic signal optical imaging and supra- and sub-threshold neuronal recordings from an array of eight independently moving electrodes as employed in previous studies of single whisker stimulation (Brett-Green et al., 2001; Frostig et al., 2008). The MWFR evoked by stimulating an array of 24 whiskers was found to possess only a single peak. This single peak was situated centrally within a large activation spread and located centrally within barrel cortex. Thus, the MWFR of 24 whiskers no longer conveyed one-to-one topographical information about the individual identities of the stimulated whiskers (e.g., 24 local peaks co-registering with the 24 appropriate whisker barrels). This main finding indicates that the rat barrel cortex response to multi-site stimulation transcends one-to-one topography, culminating in a large activation spread with a single central peak. An MWFR with a single central peak would offer a potential neurobiological mechanism for the well-known phenomenon of perceptual funneling reported across different sensory modalities and species including humans in which multi-site stimulation is perceived as though only a single, central site has been stimulated (Bekesy, 1967; Gardner and Spencer, 1972a; Gardner and Tast, 1981).

We also studied MWFRs in more detail with additional experiments, modeling, pharmacological manipulations, and comprehensive quantification. The interaction between large cortical activation spreads of individual whiskers (for review see Frostig, 2006 and Fox, 2008; for spread observed specifically beyond barrel cortex see Brett-Green et al., 2001; Ferezou et al., 2006, 2007; Frostig et al., 2008; Lim et al., 2012) was found to predict and directly contribute to the salient properties of the obtained cortical response including the single central activity peak, indicating an underlying mechanism for our MWFR findings. Compared to single whisker stimulation, the 24-whiskers MWFR peak magnitude was comparable in strength and the response area was modestly larger. Both of these response properties exhibited a reduction in variability that was particularly pronounced for the peak magnitude. Last, findings were generalized to a different set of whiskers (subgroup of 4 neighboring whiskers within the array of 24 whiskers).

## Materials and methods

Intrinsic signal optical imaging and electrophysiology recordings were performed as in previous studies and most details can be found elsewhere (Chen-Bee et al., 2000, 2007; Frostig et al., 2008). Summary and additional details are provided here.

### Subjects

All *in vivo* procedures were in compliance with the National Institutes of Health guidelines and reviewed and approved by the University of California Irvine Animal Care and Use Committee. Subjects were adult male Sprague–Dawley rats. Rats were inducted with a bolus intraperitoneal injection of sodium pentobarbital (55 mg/kg b.w.) and maintained with supplemental injections as needed throughout the day. An 8 × 8 mm region of the exposed skull centered above barrel cortex was thinned with a dental drill and kept moist with saline. Rats were then slated for one of several types of experiments differing according to method of cortical activity assessment (imaging; electrophysiology) and multi-whisker stimulation condition being studied (24- and 4-whiskers), and whether lidocaine was locally injected into the cortex.

### Whisker stimulation

**Figure 2:**
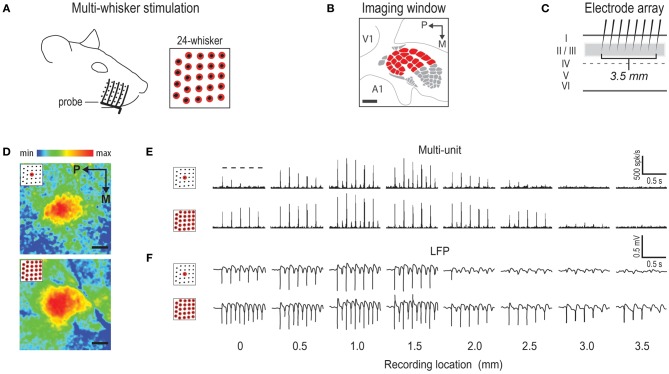
**Representative cases of rat barrel cortex response to stimulating an array of 24 whiskers. (A)** 5-prong probe used to achieve multi-whisker stimulation. Stimulated whiskers are indicated in red. **(B)** 6 × 6 mm intrinsic signal optical imaging field-of-view used when imaging activity simultaneously from the entire barrel cortex plus surrounding regions. V1 = primary visual cortex; A1 = primary auditory cortex. **(C)** Array of eight independently positioned electrodes, spaced 0.5 mm apart, used to record supra- and sub-threshold neuronal activity from cortical layers II/III. **(D)** Representative cases of imaging activity for C2 whisker (top) versus 24-whiskers (bottom). Color scale indicates fractional change in poststimulus activity relative to prestimulus activity. **(E,F)** Representative cases of supra- **(E)** and sub- **(F)** threshold neuronal activity for C2 whisker (**E,F**, top) and 24-whiskers (**E,F**, bottom). The 5 dashes indicating stimulus delivery in top panel of (**E**) apply to all supra- and sub-threshold neuronal activity panels. All provided scale bars = 1 mm. Both imaging and neuronal data are aligned according to location of peak activity for whisker C2. Note the similarity in location of peak activity and response magnitude between C2 whisker and 24-whiskers stimulation.

Whisker stimulation was restricted to only the right snout side (Figure 1A). Besides single whisker C2 stimulation, two types of multi-whisker stimulation were employed: 24- and 4-whiskers. The whiskers slated for multi-whisker stimulation were of sufficient length to allow a probe to simultaneously deflect all of them while still avoiding contact of any mystacial fur by the probe. At the start of each experiment, the presence of all 24 large whiskers in rows A–E and arcs 1–4 plus all four Greek whiskers (Figure 1A) were explicitly confirmed. All remaining (smaller) whiskers were trimmed off. As in previously established protocols, the stimulation of only whisker C2 was achieved with a copper wire probe attached to a computer-controlled stepping motor. Five deflections were delivered at 5 Hz rate for total time span of 1 s. Each deflection displaced whisker C2 approximately 1 mm along the rostra-caudal direction at a distance of approximately 5 mm from the skin. The parameters of single whisker stimulation were replicated for multi-whisker stimulation. To stimulate all 24 whiskers, a computer-controlled stepping motor was still used, except a 5-prong probe constructed by mounting five parallel copper wires spaced 2 mm apart onto a base steel rod (Figure 2A) was used instead of a single copper wire. In order for all 24 whiskers to be deflected at the same distance from the skin, the parallel copper wires were molded to follow the contour of the right snout. Also, for all 24 whiskers to be similarly displaced along the rostral-caudal direction, the resting position of the 5-prong probe was set such that all 24 whiskers were in contact with one of the five wires. The 4-whiskers stimulation was achieved in the same manner, except all but D3, D4, E3, and E4 whiskers were trimmed off prior to positioning of the 5-prong probe.

### Intrinsic signal optical imaging

Intrinsic signal optical imaging was used for high-spatial resolution, wide field-of-view mapping of the total cortical activation spread evoked by whisker stimulation; the activation spread can be referred to as a MWFR or SWFR (single whisker functional representation) depending on the number of whiskers being stimulated. Two groups of rats underwent imaging, differing according to the type of MWFR being studied, 24-whiskers (*n* = 10) or 4-whiskers (*n* = 7). In every rat, the SWFR for whisker C2 was also imaged for reference and landmark purposes. Imaging was conducted with a 16-bit CCD camera (Cascade 512B II; Photometrics, Tucson, AZ) combined with an inverted 50 mm lens plus extenders. The camera's field-of-view (Figure 2B) was a 7.42 × 7.42 mm cortical region, mapped onto a 256 × 256 pixel array. For future alignment of data files collected within the same rats as well as across rats, the field-of-view neuroaxis was oriented the same in every rat, plus the field-of-view remained constant across data files within each rat. The CCD camera was focused 600 μm below the cortical surface before the start of data collection to minimize contributions from surface blood vessels and maximize contributions integrated across the upper cortical layers. The imaged cortical region was continuously illuminated with a red LED (635 nm max, 15 nm full width at half-height). Imaging frames were captured at 10 Hz rate (i.e., 1 frame = 100 ms exposure time), and each imaging trial lasted 15 s. Onset of whisker stimulation occurred 1.5 s into the trial. A block of 64 trials was collected per whisker stimulation condition, with an intertrial interval averaging 6 s and ranging randomly between 1–11 s and thus an average of 21 s between the onset of consecutive stimulus deliveries. The 64 trials in a block were then summed and the summed data collapsed into 500-ms frames (referred to hereafter as a data file) to increase the signal-to-noise.

Imaging data files were processed and analyzed using V++ software (Digital Optics, Auckland, New Zealand). For each data file, activity for each 500-ms post-stimulus frame was converted to fractional change relative to the 500-ms frame collected immediately prior to stimulus onset on a pixel-by-pixel basis. As expected based on previous findings (Chen-Bee et al., 2007), SWFRs of whisker C2 typically consisted of an imaging signal spanning 10+ s that was triphasic in nature (initial dip below baseline followed by a large overshoot and large undershoot; see Supplementary Presentation 1, middle panel). Within the same subjects, MWFRs were observed to also consist of a triphasic signal; see Supplementary Presentation 1, bottom panel. Detailed analysis of every imaging data file was restricted to the first signal phase (initial dip). The first 500-ms frame containing the maximum areal extent of evoked initial dip activity was processed with a two-pass Gaussian filter (half-width = 5) to remove high-frequency spatial noise. Filtered values were subsequently used for all plotting, quantification, and statistics performed with MATLAB and SYSTAT.

Alpha level was set to 0.05 for all statistical tests performed on the imaging results. For every data file, the location and magnitude of peak activity were obtained from the pixel with the greatest magnitude within the evoked activity area. Areal extent of the evoked activity area was quantified using a constant threshold of −2.5 × 10^−4^ FC, or −0.025%, away from 0 (approximately half-max). The peak magnitude and areal extent of the evoked activity area obtained from each MWFR data file was compared to those for single whisker C2 obtained in the same rats using two-tailed paired *t*-tests. For remaining statistics see Table 1.

**Table 1:** **Statistics summaries of multi-whisker functional representation (MWFR) and single whisker functional representation (SWFR) data**.

1	See Figure 3C (24-whiskers vs. C2). For the 24-whiskers array, the imaging values were compared between the *in vivo*-MWFR and whisker C2's *in vivo*-SWFR obtained within each of 10 rats. Comparison was restricted to the rostral-caudal slice through the center of C2 barrel, corresponding also through the center of the MWFR as well as the SWFR. A two-way repeated measures ANOVA was performed on the imaging values, with the two main variables being Cortical Location (coordinates along the rostral-caudal slice) and Activity Type (MWFR vs. SWFR). Imaging values were first log-transformed to satisfy ANOVA assumptions. The interaction between the two main variables, Cortical Location and Activity Type, was found significant [*F*_(220, 1980)_ = 4.18, *p* = 9.99 × 10^−16^], supporting the observed differences in the relationship between the MWFR and the SWFR magnitudes depending on the cortical location along the rostral-caudal slice (e.g., no difference at the location of peak activity while larger magnitudes for the MWFR at distances away from the peak).
2	See Figure 3D. For the 24-whiskers array, the magnitude of underlying sub-threshold neuronal activity was compared between the MWFR and whisker C2's SWFR obtained within each of 12 rats. Comparison was made for 8 electrodes recording simultaneously from the cortex and spaced 0.5 mm apart along the tangential plane. Electrodes were positioned such that activity could be sampled on opposite sides of the C2 barrel as well as increasing distances away. In support of data observations and congruent with Summary 1 above, a two-way repeated measures ANOVA performed on the log-transformed values found the interaction between Cortical Location and Activity Type significant [*F*_(6, 66)_ = 10.54; *p* = 3.53 × 10^−8^].
3	See Figure 3E. Same as Summary 2 above, except for the magnitude of supra-threshold neuronal activity. A two-way repeated measures ANOVA found the interaction between Cortical Location and Activity Type significant [*F*_(6, 66)_ = 12.65; *p* = 1.92 × 10^−9^].
4	See Figure 4F. For the 24-whiskers array, the goodness-of-fit of the imaging magnitude values was measured between the model-MWFR (Figure 4D) and the set of *in vivo*-MWFRs obtained from 10 rats (Figure 4E). The model-MWFR was first normalized to the same peak magnitude as the *in vivo*-MWFR. Then, reduced chi-squared tests were performed on a pixel-by-pixel basis within a 4.12 × 2.75 mm cortical region comprising the barrel cortex associated with the 24 largest whiskers plus nearby surrounding regions. A reduced chi-squared value of 1.125 indicated the best fit possible as achieved by using the mean of the 10 rats. Obtained chi-squared values ranged 1.125-8.536; mean ± SD = 1.925 ± 0.846.
5	See Figure 4F. Same as Summary 4 above, except the *in vivo*-MWFR values were averaged across the 10 rats before comparison to the values of the normalized model-MWFR using a least-squares linear regression. An R-Squared value = 1 indicated that 100% of the variance in the average *in vivo* values across the 4.12 × 2.75 mm cortical region could be explained by the model. Obtained R-Squared value = 0.80. A least-squares linear regression to the model as defined with an incorrect set of SWFRs (specifically the one in Figure 6G) resulted in an R-Squared value = 0.09.
6	See Figures 5D,E. Two-Way repeated measures ANOVA was performed on the sub-threshold response magnitude values (log-transformed to satisfy ANOVA assumptions), with the two main variables being Cortical Location (8 electrode recordings spaced 0.5 mm apart) and Recording Condition (before vs. after lidocaine injection) (see Figure 5A). The interaction between Cortical Location and Recording Condition was found significant [*F*_(7, 28)_ = 10.95, *p* = 1.39 × 10^−6^], indicating that differences between recording conditions were dependent on cortical location and supporting the obtained results of decreased response magnitude for cortical locations within the infusion site and increased response magnitude for locations outside the infusion site (Figure 5D). Supra-threshold response magnitudes (Figure 5E) underwent the same analysis and complementary findings were obtained in which a significant interaction was also found between Cortical Location and Recording Condition [*F*_(7, 28)_ = 9.68, *p* = 4.48 × 10^−6^].
7	See Figure 6B. Same as described for the 24-whiskers array in Summary 1 above, for whiskers D3D4E3E4 the absolute imaging values were compared between the *in vivo*-MWFR and whisker C2's *in vivo*-SWFR obtained within each of 7 rats. A Two-Way repeated measures ANOVA performed on the log-transformed values found the interaction between the main variables Cortical Location and Activity Type significant [*F*_(151, 906)_ = 1727.69, *p* = 9.99 × 10^−16^), supporting the observed differences in the relationship between the MWFR and SWFR magnitudes once location along the rostral-caudal slice is taken into consideration (e.g., MWFR is larger at some locations but smaller at other locations).
8	See Figure 6C. Same as described for the 24-whiskers array in Summary 2 above, for whiskers D3D4E3E4 the magnitude of underlying sub-threshold neuronal activity was compared between the MWFR and whisker C2's SWFR obtained within each of 10 rats. Positioning of the 8 electrodes within the cortex was optimized to detect the shift in peak location between the MWFR and the SWFR. In support of data observations and congruent with Summary 7 above, a Two-Way repeated measures ANOVA performed on the log-transformed values found the interaction between Cortical Location and Activity Type significant [*F*_(6, 48)_ = 20.67, *p* = 8.64 × 10^−12^].
9	See Figure 6D. Same as Summary 8 above, except for the magnitude of supra-threshold neuronal activity. A Two-Way repeated measures ANOVA found the interaction between Cortical Location and Activity Type significant [*F*_(6, 48)_ = 6.73, *p* = 3.00 × 10^−5^].
10	See Figure 6H. Same as described for the 24-whiskers array in Summary 4 above, for whiskers D3D4E3E4 the goodness-of-fit was measured between the model- (Figure 6G) and the set of *in vivo*-MWFRs obtained from 7 rats (Figure 6A). A reduced chi-squared value of 1.200 indicated the best fit possible, with obtained values ranged 1.200–4.574; mean ± SD = 1.629 ± 0.434.
11	See Figure 6H. Same as Summary 10 above, except the *in vivo*-MWFR values were averaged across the 7 rats before comparison to the model-MWFR normalized values using a least-squares linear regression. Obtained R-Squared value = 0.79. Least-squares linear regression to the model as defined with an incorrect set of SWFRs (specifically the one in Figure 4D) resulted in an R-Squared value = 0.26.

**Figure 3:**
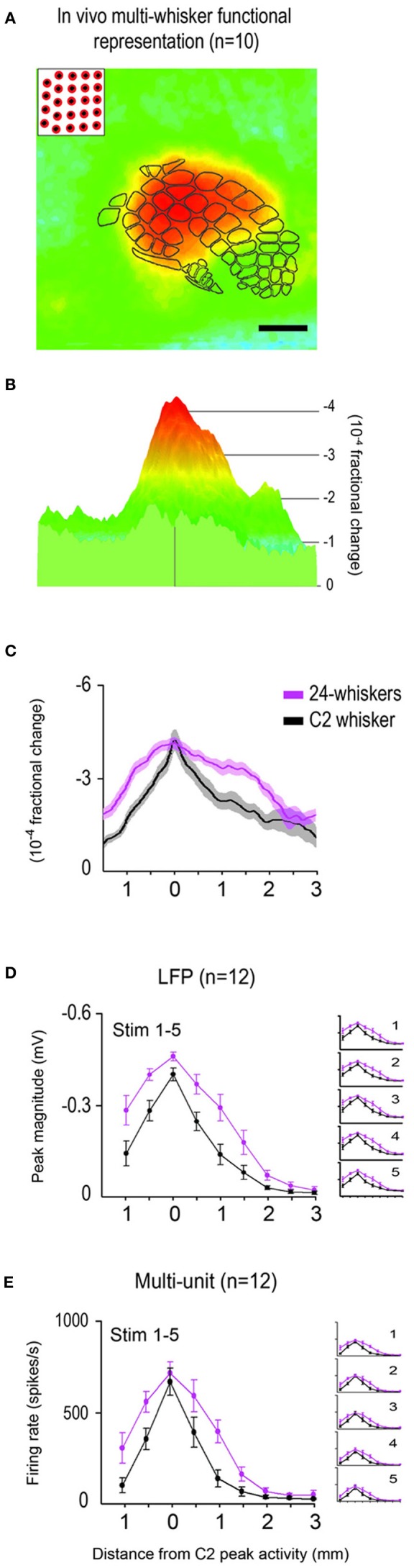
**Average *in vivo* data for the 24-whiskers array.** The multi-whisker functional representation (MWFR) for the 24-whiskers array was assessed *in vivo* using intrinsic signal optical imaging (*n* = 10; **A–C**) and supra- and sub-threshold neuronal recordings from an 8-electrode array (*n* = 12; **D,E**). The single whisker functional representation (SWFR) for whisker C2 was also assessed in the same rats for reference. **(A–C)** The average *in vivo*-MWFR for the 24-whiskers as assessed with imaging for the same 6 × 6 mm field-of-view can be plotted in 2D with barrel cortex topography superimposed **(A)** or in 3D **(B)**. It can also be plotted as a line plot for the rostral-caudal slice through the center of whisker C2 barrel and hence through the center of the 24-whiskers' MWFR and C2 whisker's SWFR (**C**; mean ± SE as solid line and shading, respectively). Scale bar = 1 mm in **(A)**; colorscale is the same in **(A,B)**. (**D,E**) The average *in vivo*-MWFR as assessed with neuronal recordings. Plotted is the mean ± SE of sub- **(D)** and supra- **(E)** threshold neuronal activity of the MWFR for 24-whiskers (magenta trace) vs. the SWFR for whisker C2 (black trace). Whisker stimulation consisted of 5 back-and-forth whisker deflections in the rostral-caudal direction delivered at 5 Hz; parent panels contain data averaged across the five stimulus whisker deflections whereas panel insets contain data separated according to stimulus deflection. For both imaging (**A–C)** and neuronal recording **(D,E)** data, note that the MWFR for the 24-whiskers array consists of a single peak located centrally within a large activation spread, thus resembling a relatively symmetrical activity mountain with one peak. Also note that, compared to the single whisker C2, the 24-whiskers' MWFR exhibited no shift in location of peak activity, no **(C,E)** or modest (14%; **D**) increase in peak magnitude, and relatively moderate increases in the tangential spread of activity and thus a broader shape of the activity mountain.

To permit 2D and 3D plotting of averages (Figures 3A,B, respectively), as well as statistical testing, on a pixel-by-pixel basis, filtered data across rats were first aligned in the following manner. Because the spatial scale and neuroaxis were the same for all data files, along with known findings of a single whisker's peak activity co-registering with the appropriate topographical location within barrel cortex (e.g., that whisker's barrel), the whisker C2's peak location identified for each rat was used for aligning data across rats. Whisker C2 peak location was used irrespective of the type of data being aligned (C2, 24- or 4-whiskers) as the field-of-view remained constant across different data files within the same animal. Aligned data were used not only for plotting of average data across rats, but also for various statistical comparisons between stimulation of single whisker C2 (within subjects reference data) vs. 24-whiskers (Table 1, Summary-1) or 4-whiskers (Table 1, Summary-7), or statistical comparisons to modeled data (Table 1, Summaries 4–5 and 10–11).

### Electrophysiology

Two groups of rats underwent electrophysiology recordings, analogous to imaging, in which they differed according to the type of MWFR being studied: 24-whiskers (*n* = 12) or 4-whiskers (*n* = 9). In every rat, the SWFR for whisker C2 was also assessed. Craniotomy and dura removal were performed above barrel cortex and surrounding cortical regions, and the cisterna magnum drained of cerebrospinal fluid to minimize edema and brain pulsation. As in previous studies (Frostig et al., 2008), imaging of whisker C2's SWFR was first performed so that the peak activity location could be used to guide placement of electrodes [imaging peak location overlies C2 barrel (Masino et al., 1993; Brett-Green et al., 2001)]. Subsequently, simultaneous recordings were obtained from eight cortical locations spanning 3.5 mm along the cortical tangential plane with the use of eight Tungsten microelectrodes (~1.5 MΩ impedance; MicroProbe Inc., MD, US; Figure 2C). Electrodes were spaced 0.5 mm apart and linearly aligned, and were independently inserted into the cortex perpendicularly to the cortical surface using a micropositioner (EPS, Alpha-Omega, Nazareth, Israel). Recording depth was ~400 μm below the cortical surface corresponding to supragranular layers II/III. Placement of the eight electrodes was optimized according to the type of experiment being pursued, within the constraints of large cortical surface blood vessels. For 24-whiskers stimulation experiments, the second or third electrode was aimed at whisker C2's imaging peak location in order to permit recordings on either side of peak activity while still allowing recordings at far distances away from the peak (toward the medial-caudal direction). For 4-whiskers stimulation experiments, the middle electrodes were aimed at whisker C2's imaging peak location and the eight electrodes aligned parallel to the rostral-caudal axis in order to best detect a shift in peak activity location. Recorded signal was amplified and filtered on-line to allow simultaneous capture of supra-threshold (multi-units; 300–3000 Hz bandpass) and sub-threshold (local field potentials or LFPs; 150 Hz low pass) neuronal activity, and then digitized at 24 KHz rate. Quality and consistency of recordings were monitored throughout the day with real time assessment of multi-unit and LFP signals from every electrode. As with imaging, a block of 64 stimulation trials were collected per stimulation condition, contained within one continuous data trace per electrode and with consecutive stimulus deliveries occurring 21 s apart on average.

All off-line analysis including quantification, plotting, and statistics were performed using Spike 2 software (CED, Cambridge, England), MATLAB, and SYSTAT. For each block of 64 stimulation trials, the recorded data from each of the 8 electrodes were analyzed in the same manner. The LFP data were averaged across trials and the peak magnitude (first minimum) determined for each of the five whisker deflections comprising a complete stimulus delivery. The peak magnitudes could then be averaged together or separated for subsequent comparisons between whisker stimulation conditions. For the multi-unit data, spiking events were qualified with a threshold criterion (±3 SD away from the mean calculated from the entire data trace excluding outliers), averaged across trials in 5 ms bins, and expressed as firing rate per second per trial before proceeding in the same manner as for the LFP analysis.

Statistical tests are described in Table 1. Alpha level was set to 0.05 for all statistical tests performed on the electrophysiological results.

### Modeling multi-whisker functional representations (MWFRs) based on linear summation of single whisker functional representations (SWFRs)

**Figure 4:**
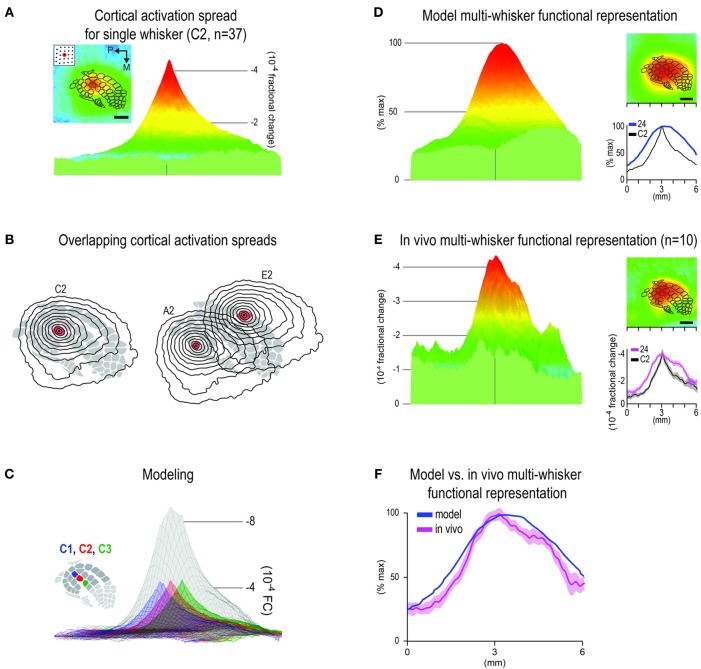
**Predicting the *in vivo* multi-whisker functional representation (MWFR) for the 24-whiskers array based on linear summation of single whisker functional representations (SWFRs). (A)** Average activity in barrel cortex evoked by a single whisker plotted in 2D with barrel cortex topography superimposed (inset; scale bar = 1 mm) or in 3D (parent panel), both for the same 6 × 6 mm cortical region. Based on 37 rats assessed with intrinsic signal optical imaging, this example serves as the representative SWFR used in modeling the cortical response to many whiskers. Note the ability of a single whisker to evoke a large spread of cortical activity spanning across many barrels. **(B)** Contour plots (isolevels spanning from −1 to −4 × 10^−4^ fractional change in increments of 0.25 × 10^−4^) of the SWFR for whisker C2 only (left) and whiskers A2 and E2 (right) superimposed on barrel cortex topography. Note the large amount of spatial overlap between cortical activation spreads even for whiskers whose barrels are at opposite borders of barrel cortex. **(C)** Modeling the MWFR based on linear summation of SWFRs. A simple example is provided here, in which three copies of the representative SWFR are aligned according to C1 (blue), C2 (red), or C3 (green) barrel location before their linear summation to generate the model-MWFR for whiskers C1–C3 (gray). **(D–F)** Model- vs. *in vivo*-MWFR for the 24-whiskers array. **(D)** Model-MWFR for 24-whiskers for same 6 × 6 mm field-of-view plotted in 3D (parent panel) or 2D (top inset), or as a line plot for the rostral-caudal slice through the center of C2 whisker barrel (bottom inset), blue trace; black trace is for the representative SWFR in **(A)**. **(E)** Average *in vivo*-MWFR for 24-whiskers in Figures 3A–C is shown here for easier comparison to the model-MWFR provided in **(D)**. Note that the model-MWFR exhibited many of the salient properties observed for the *in vivo*-MWFR: symmetrical activity mountain with one central peak; peak location aligned with that for whisker C2 and thus located centrally within barrel cortex; a relatively broader mountain shape compared to whisker C2. **(F)** Response magnitudes for the rostral-caudal slice through the center of whisker C2 barrel are plotted to illustrate the goodness-of-fit between the normalized model- vs. *in vivo*-MWFR.

We empirically derived an SWFR with representative properties (topography; peak magnitude; signal decay over distance) based on imaging data collected across several intrinsic signal optical imaging projects including the present study (*n* = 37 rats). This set of data shared the same surgical and data acquisition protocols for imaging the single whisker C2, and the same data processing up through spatial filtering, as in the present study. Because peak activity of a single whisker co-localizes above that whisker's appropriate anatomical barrel (Masino et al., 1993; Brett-Green et al., 2001; Frostig et al., 2008), the filtered images from the 37 rats were spatially aligned according to peak activity location before averaging across images. The resultant average image served as the representative SWFR with empirically derived representative properties including peak magnitude and signal profile (Figures 4A,B). Then, models of MWFRs were generated by (1) creating the appropriate number of copies of the representative SWFR; (2) spatially aligning those copies according to barrel cortex topography; and (3) linearly summating the aligned copies. For a simplified example of modeling see Figure 4C.

### Electrophysiology experiments with local silencing of cortical activity

A last set of electrophysiology experiments (*n* = 5 rats) were conducted to investigate the role played by local cortical activity. These experiments were conducted and analyzed in the same manner as the original set of electrophysiology experiments (see Methods Section “Electrophysiology”) except: (1) multi-whisker stimulation was restricted to the 24-whiskers array; (2) middle electrodes of the 8-electrode array were inserted into the location of peak cortical activity; and (3) data acquisition occurred before and after lidocaine was injected locally into the cortex. 1 μL of lidocaine (10%; Sigma) was slowly microinjected over the course of 3 min at 300–450 microns cortical depth between the first and second electrode (thus 1.5 mm distal from the middle electrode corresponding to peak activity location). Lidocaine injection followed a previously used protocol (Frostig et al., 2008) in which the lateral spread of lidocaine injection was deemed less than 1 mm in radius away from the injection site. Three sessions of data collection were collected in every rat, initiated before, few minutes after, and one hour after lidocaine injection.

## Results

### Multi-whisker functional representation (MWFR) of 24 whiskers possesses a single central peak

The MWFR of the 24 largest whiskers (array of neighboring whiskers located in rows A–E and arcs 1–4, plus the four Greek whiskers; Figure 1A) was mapped using intrinsic signal optical imaging (*n* = 10 rats) with a wide imaging field-of-view (Figure 2B). In all rats, the SWFR of whisker C2 was also imaged for reference. Representative and average imaging data are provided in Figure 2D and Figures 3A–C, respectively.

As expected, on average the SWFR evoked by stimulating whisker C2 consisted of a single activity peak surrounded by a large spread of decaying activity, culminating in a response profile that resembled a single peaked and relatively symmetrical mountain of activity (see black trace in Figure 3C). When the number of whiskers being stimulated increased from 1 to 24, the average MWFR for the 24-whiskers still possessed only a single activity peak (Figures 3A–C), thereby no longer conveying topographical information about the individual identities of the stimulated whiskers (e.g., 24 local peaks co-registered with the stimulated whisker barrels). This single peak was at a similar location as for whisker C2's activity peak (in Figure 3C, compare black trace of C2 whisker to magenta trace of 24-whiskers), meaning that the peak was located centrally within barrel cortex (Figure 3A). Note also that this meant the peak location was situated centrally within the collection of the 24 stimulated whiskers' individual SWFRs. Last, compared to whisker C2, there was no increase in the magnitude of peak activity, and the decay of activity away from the peak was more gradual, resulting in an activity mountain with a broader shape (Figure 3C). See Table 1, Summary 1, for statistical results obtained from a two-way repeated measure ANOVA performed on the intrinsic signal optical imaging data to compare between the 24-whiskers array's MWFR and whisker C2's SWFR.

The MWFR of the 24-whiskers array, along with whisker C2's SWFR, was also assessed with electrophysiology recordings in each of 12 rats (representative and average data provided in Figures 2E,F and Figures 3D,E, respectively). Congruent electrophysiology results for the 24-whiskers array were obtained for both sub- and supra-threshold neuronal activity (parent panels in Figures 3D,E, respectively; Table 1, Summaries 2–3, respectively) in which: (1) a single activity peak was still observed; (2) that was in the same location as for whisker C2 and hence located centrally within barrel cortex; (3) with modest (14% for sub-threshold) or no (supra-threshold) increase in magnitude compared to whisker C2; and (4) surrounded by a spread of activity that decayed more gradually away from the peak compared to whisker C2. Additionally, while the sub- and supra-threshold results provided in the parent panels of Figures 3D,E were for the average response across the five stimulation pulses delivered at 5 Hz rate, we observed the same results when data were subdivided according to stimulation pulse (see insets in Figures 3D,E).

### Salient properties of the 24-whiskers' multi-whisker functional representation (MWFR) can be predicted by, and is dependent on, interaction between single whisker functional representations (SWFRs) of individual whiskers

Based on the well-known topographical and columnar organizational principles of cortical function (Figure 1), we could not readily account for the finding of a single activity peak for the 24-whiskers' MWFR (Figure 3). Thus, we pursued modeling and pharmacological experiments to determine whether a single whisker's ability to evoke a large cortical activation spread spanning across many barrels (Figure 4A) might offer some explanation. As illustrated in Figure 4B, the large tangential spread of any given SWFR ensures substantial overlap in the cortical territory occupied by SWFRs of different whiskers even when they are far apart. This overlap should be conducive for SWFRs to interact with one another. We investigated whether interacting SWFRs contributed to the final cortical response evoked by stimulating many whiskers together.

We generated a model of the MWFR for the 24-whiskers array to be expected if cortical activity were indeed dependent on the stereotypical properties of SWFRs and the simplest form of interaction between them (linear summation; Figure 4C). The SWFR of whisker C2 as averaged across 37 rats served as the representative SWFR for any large whisker. This representative SWFR possessed empirically derived properties such as a peak magnitude of −4 × 10^−4^ fractional change and a specific signal decay function away from the peak. The generated model-MWFR for the 24-whiskers array is shown in Figure 4D. We found that modeling based on linear summation of SWFRs was successful in predicting many salient properties of the MWFR observed *in vivo*. Same as for the *in vivo*-MWFR (Figure 4E, parent panel), the model-MWFR (Figure 4D, parent panel) also possessed only a single activity peak. Also, as observed *in vivo* (Figure 4E, insets), the single peak of the model-MWFR was at a similar location to whisker C2's activity peak (Figure 4D, lower inset), thus centrally located within barrel cortex (Figure 4D, upper inset) and centrally situated within the collection of the 24 individual SWFRs. After normalizing the model-MWFR to the same peak magnitude as the *in vivo*-MWFR (the entire model-MWFR was divided by a constant term of 12.8), the model-MWFR was observed to be relatively broader in shape as compared to a single whisker (Figure 4D, lower inset), which was also observed *in vivo* (Figure 4E, lower inset). Indeed, a high goodness-of-fit for the overall mountain profile (including peak location) was found between the model-MWFR and the *in vivo*-MWFR (Figure 4F; Table 1, Summary-4). The model accounted for 80% of the *in vivo*-MWFR variance across cortical location, which was greatly reduced to 9% if the model was defined using an incorrect set of SWFRs (Table 1, Summary-5).

**Figure 5:**
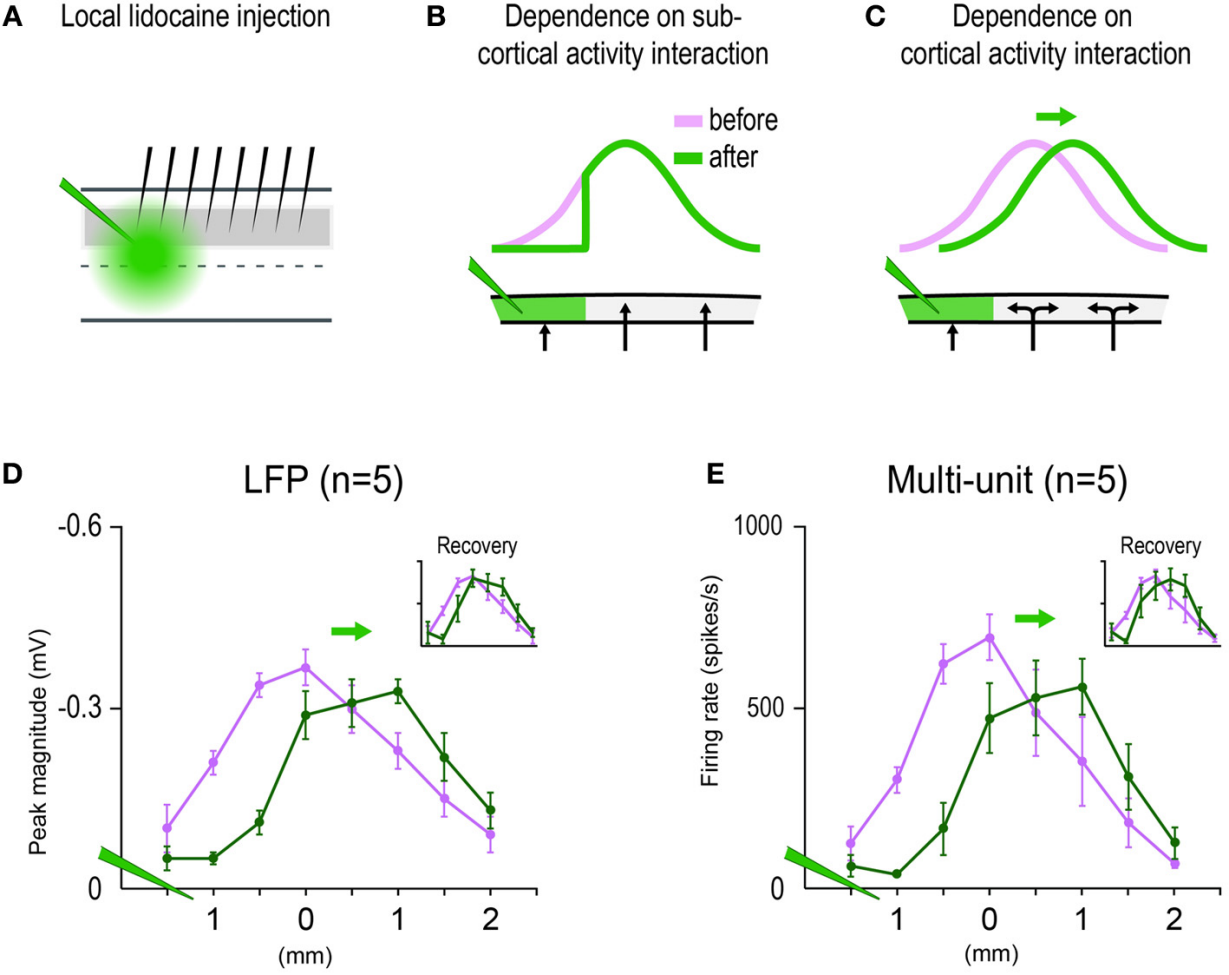
**Multi-whisker functional representation (MWFR) for the 24-whiskers array dependent on single whisker functional representation (SWFR) interactions occurring at the cortical level. (A)** Eight electrodes spaced 0.5 mm apart recorded the MWFR for 24-whiskers (middle electrodes aimed at MWFR peak activity location) before and after local lidocaine injection (green) deposited distal (1.5 mm) to the middle electrodes. 1 μL of lidocaine (10%; Sigma) was slowly microinjected over the course of 3 min at 300–450 μm cortical depth. **(B)** If sub-cortical activity interactions are the sole contributors to the MWFR, then only local silencing of cortical activity within the lidocaine site would occur which, in turn, would lead to a decrease in MWFR response magnitude only within the lidocaine site and thus no shift in MWFR peak activity location nor increases in MWFR response magnitude would occur outside the lidocaine site. **(C)** If SWFR interactions indeed occur at the cortical level (double-headed arrows), then not only the silencing of cortical activity would be induced within the lidocaine site, but also the disruption of SWFR interactions leading to a new spatial distribution of overlapping activity across unaffected SWFRs as though stimulating only a subset of the 24 whiskers. The local silencing of cortical activity should lead to the expected decrease in MWFR response magnitude within the lidocaine site while, importantly, the new SWFR activity overlap should lead to a shift in MWFR peak location away from the lidocaine site and even an increase in MWFR response magnitude outside the lidocaine site. **(D,E)** Results from sub- **(D)** and supra- **(E)** threshold neuronal recordings initiated before versus few minutes after targeted lidocaine injection are congruent with predictions based on SWFR interactions occurring at the cortical level. Recordings initiated 1 h after lidocaine injection revealed recovery of response almost to pre-injection levels (insets).

Additional experiments were conducted to explicitly verify that the *in vivo*-MWFR and its salient properties such as a single central peak are indeed dependent on interactions between SWFRs. These experiments also addressed whether SWFR interactions responsible for the single-peaked MWFR at the very least occurred at the cortical level, as opposed to subcortical interactions being solely responsible. For example, a single-peaked activity mountain could have already been established subcortically and then passively transmitted to the cortex. Additional 24-whisker MWFR electrophysiological experiments (*n* = 5 rats) were conducted in which cortical activity was locally silenced by injecting lidocaine into the cortex distal to the MWFR peak location (Figure 5A). If the MWFR depends on SWFR interactions that occurred at the cortical level, then the MWFR would alter in ways not easily explained by local silencing of cortical activity. Not only would lidocaine induce the expected decrease in activity at cortical locations within the infusion site, but it would also disturb SWFR interactions occurring within the infusion site. A new spatial distribution of overlapping activity across SWFRs (as though stimulating only a subset of the 24 whiskers) would be expected, which in turn would lead to a shift in peak location of the MWFR away from the injection site and even an increase in MWFR activity magnitude outside the infusion site (Figure 5C). In contrast, if sub-cortical interactions were the sole contributors, this local silencing of cortical activity would not reduce the magnitude of peak activity or shift its location. Instead, a decrease in MWFR activity magnitudes would occur merely at cortical locations within the lidocaine infusion site (Figure 5B). As seen in Figures 5D,E, local silencing of cortical activity succeeded in shifting the location of peak activity away from the injection site and increasing the activity magnitude at locations outside the lidocaine infusion site for both sub- (Figure 5D) and supra- (Figure 5E) threshold neuronal activity. See Table 1, Summary-6.

### Multi-whisker functional representation (MWFR) findings extend to a different combination of neighboring whiskers

Additional *in vivo* and modeling experiments were conducted to determine whether findings could be extended to a different combination of neighboring whiskers (the four whiskers D3, D4, E3, and E4). Note these four whiskers are located off-center within the 24-whiskers array (refer to Figure 1A) and hence the center of their individual SWFRs is located rostral to the center of barrel cortex (refer to Figure 1B).

**Figure 6:**
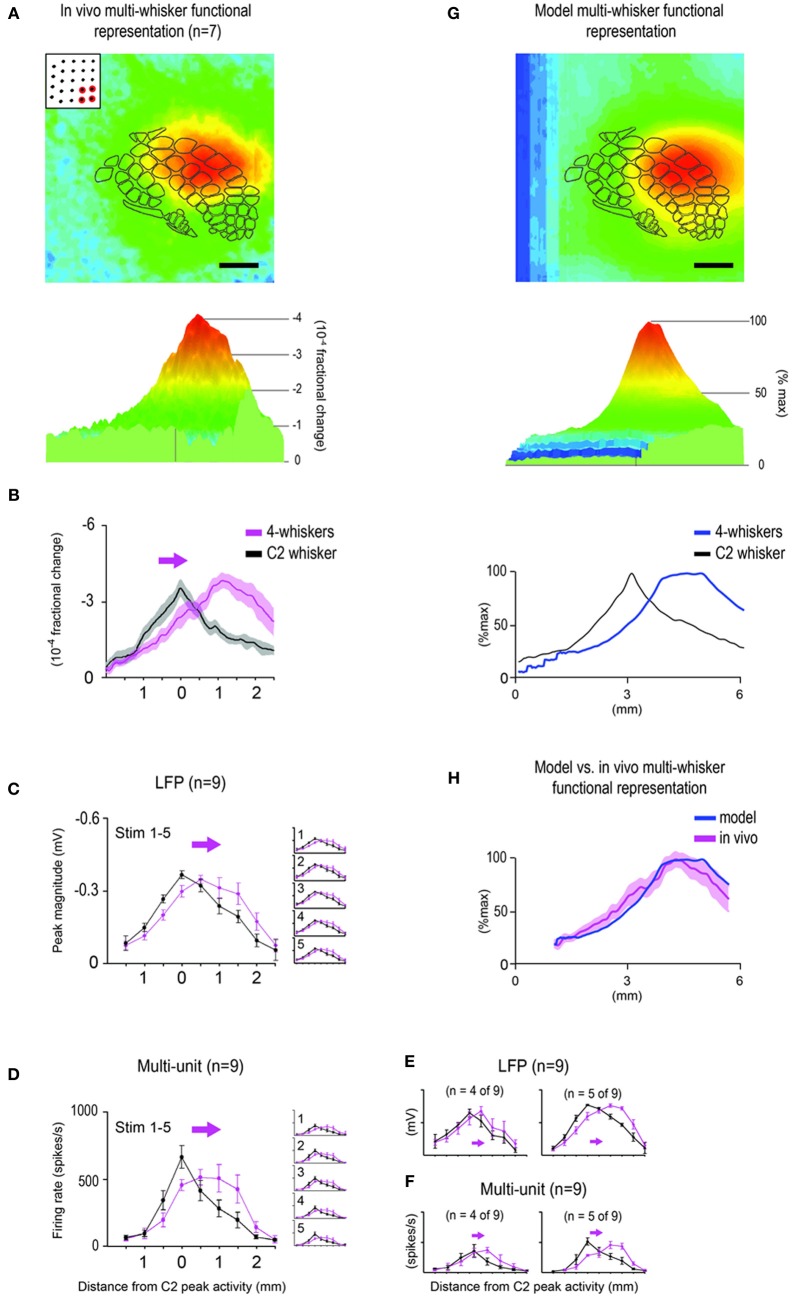
**Subset of 4 whiskers also evokes an activity mountain with a single central peak that can be predicted by modeling.** The multi-whisker functional representation (MWFR) for the 4-whiskers array (D3, D4, E3, and E4) was assessed *in vivo* using intrinsic signal optical imaging (*n* = 7; **A–B**) and supra- and sub-threshold neuronal recordings from an 8-electrode array (*n* = 9; **C–F**), and compared to modeling based on linear summation of single whisker functional representations **(G–H)**. Details are the same as described in Figures 3–4 except the MWFR is for 4-whiskers instead of 24-whiskers. Note for both imaging **(A,B)** and neuronal recording **(C,D)** data, the MWFR for the 4-whiskers array consisted of a single peak located centrally within a large activation spread, thus resembling a relatively symmetrical activity mountain with one peak. Also, compared to the single whisker C2, the 24-whiskers' MWFR exhibited a shift in location of peak activity (see arrow in **B–D**), similar peak magnitude, and similar tangential spread of activity and thus a similarly broad mountain of activity. Although the activity mountain for the 4-whiskers appeared less peaked compared to C2 whisker, particularly for the supra-threshold activity **(D)**, once sub- **(C)** and supra- **(D)** threshold neuronal activity for 4-whiskers were subdivided into two groups according to whether the peak location for 4-whiskers shifted by one (*n* = 4 out of 9 rats; left panels in **E,F**) or two (*n* = 5 out of 9 rats; right panels in **E,F**) electrode recording locations, the spatial profile for 4-whiskers no longer appeared less peaked compared to whisker C2. Last, the model-MWFR exhibited many of the salient properties observed for the *in vivo*-MWFR: symmetrical activity mountain with one central peak (**G**, middle panel), peak location shifted away from that for whisker C2 (**G**, bottom panel) and thus located off-centered within barrel cortex (**G**, top panel), a relatively similar broad activity mountain compared to whisker C2 (**G**, bottom panel), ultimately leading to a high goodness-of-fit with data obtained *in vivo* **(H)**.

The *in vivo*-MWFR for the 4-whiskers exhibited many properties similar to the 24-whiskers. As assessed with intrinsic signal optical imaging (*n* = 7 rats), the *in vivo*-MWFR for the 4-whiskers also consisted of a symmetric activity mountain with one central peak (Figure 6A, bottom panel). Same as for the 24-whiskers, the 4-whiskers activity peak was situated centrally within the stimulated whiskers' individual SWFRs. Unlike for the 24-whiskers, however, the 4-whiskers activity peak no longer resided in the same location as whisker C2's peak (Figure 6B). Rather, it was shifted toward the rostral direction and hence no longer located centrally within barrel cortex (Figure 6A, top panel). As for the 24-whiskers, the peak magnitude for the 4-whiskers did not increase compared to whisker C2 (Figure 6B). The decay of activity away from the peak, however, was more similar to whisker C2 (Figure 6B), which contrasted with the more gradual decay observed for the 24-whiskers (Figure 3C). See Table 1, Summary-7. Last, results from electrophysiology experiments (*n* = 9 rats; Figures 6C,D; Table 1, Summaries 8–9) once again corroborated the intrinsic signal optical imaging results. With respect to underlying neuronal activity (particularly for supra-threshold), the overall signal decay profile for the 4-whiskers appeared somewhat weaker and less peaked in shape than expected (Figures 6C,D, magenta traces) based on intrinsic signal optical imaging results obtained *in vivo* (Figures 6A,B). Upon further inspection, the electrophysiology data set from 9 rats was found to consist of two subsets. The peak location was found to have shifted one electrode recording location (0.5 mm) for one set (*n* = 4 out of 9; Figures 6E,F, left plot, magenta traces) and two recording locations (1.0 mm) for the other set (*n* = 5 out of 9; Figures 6E,F, right plot, magenta traces). After their separation, the spatial activity profiles no longer appeared weaker or less peaked compared to single whisker C2's SWFR (Figures 6E,F, black traces).

Once again, modeling based on linear summation of SWFRs was successful in predicting many salient properties of the MWFR observed *in vivo*, this time for a different group of neighboring whiskers: (1) single activity peak (Figure 6G, middle panel); (2) this single peak was located centrally within the stimulated whiskers' individual SWFRs but rostral to the center of barrel cortex (Figure 6G, top panel) and thus rostral to whisker C2's peak location (Figure 6G, bottom panel); and (3) shape of activity mountain similar in broadness to whisker C2 (Figure 6G, bottom panel). A high goodness-of-fit for the overall mountain profile was found between the model-MWFR and the *in vivo*-MWFR (Figure 6H; Table 1, Summary-10), with 79% of the *in vivo*-MWFR variance across cortical location explained by the model that greatly reduced to 26% if the model was defined using an incorrect set of SWFRs (Table 1, Summary-11).

### Multi-whisker functional representation (MWFR) response properties obtained *in vivo* exhibit reduction in variability

**Figure 7:**
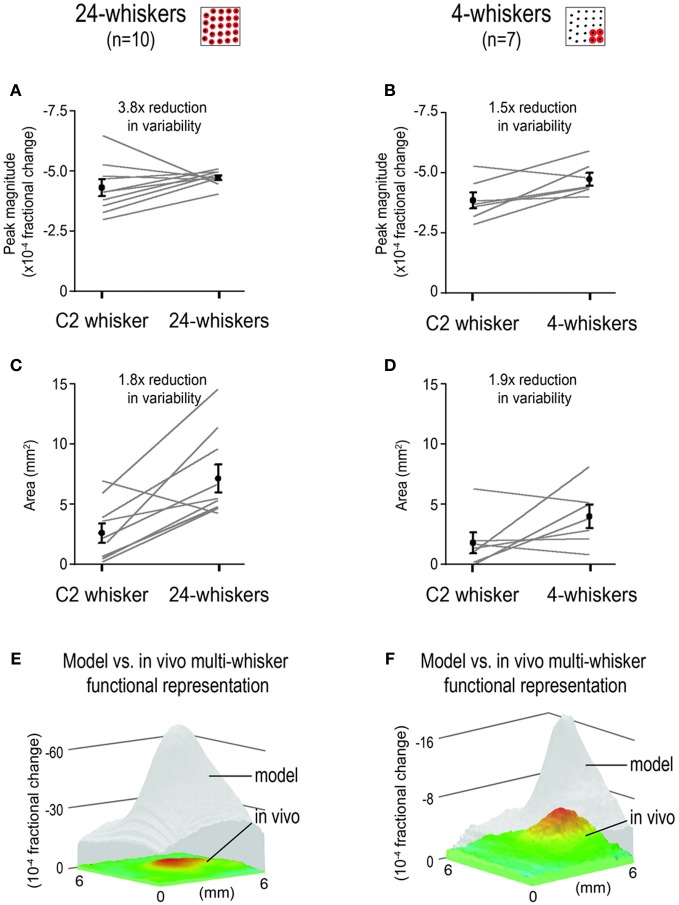
**Reduction in variability of response properties for multi-whisker functional representations (MWFRs) compared to single whisker functional representation (SWFR).** Individual and mean ± SE values of MWFR peak magnitude **(A,B)** and area **(C,D)** for 24-whiskers **(A,C)** and 4-whiskers **(B,D)** are provided. For both sets of rats, values for whisker C2 SWFR are obtained within the same animals. Area is quantified using a constant threshold of 2.5 × 10^−4^ fractional change; peak magnitude is from the pixel location with the peak activity. The coefficient of variation (ratio between mean and variance) is used as a measure of degree in response variability. Note the reduction in response variability for the MWFRs as compared to the SWFR for whisker C2, particularly apparent for the peak magnitude of the 24-whiskers MWFR in panel **(A)**. **(E,F)** Activity mountains plotted on the same z-scale range to illustrate that the MWFR obtained *in vivo* (color) is much weaker than predicted by modeling based on simple linear summation (transparent gray).

Additional analysis was performed on the *in vivo*-MWFR response properties for the 24- and 4-whiskers, specifically the intrinsic signal optical imaging magnitude at peak activity location and the areal spread of activity quantified using a constant activity threshold (Figure 7). For the 24-whiskers, the average peak magnitude was no different (two-tailed paired *t-test, t*_(9)_ = 1.21, *p* = 0.26; Figure 7A) and the average activity area was 2.6x greater (two-tailed paired *t*-test, *t*_(9)_ = 4.16, *p* = 0.002; Figure 7C) compared to those for single whisker C2. Of particular interest was the observed reduction in variability of the *in vivo*-MWFR response properties as measured by the coefficient of variation (ratio between SD and mean)—3.8x reduction for the peak magnitude (Figure 7A) and 1.8x reduction for the activity area (Figure 7C). For the 4-whiskers, the peak magnitude was found to be significantly but modestly stronger (1.2x; two-tailed paired *t*-test, *t*_(6)_ = 2.72, *p* = 0.03; Figure 7B) while the activity area was found not significantly different (two-tailed paired *t-test, t*_(6)_ = 1.86, *p* = 0.11; Figure 7D) compared to whisker C2. Again, a reduction in variability was observed, 1.5x for the peak magnitude (Figure 7B) and 1.9x for the activity area (Figure 7D), although it was less pronounced compared to that observed for the 24-whiskers.

As illustrated in Figures 7E,F, the absolute magnitude values of the *in vivo*-MWFR were much weaker than predicted by modeling based on linear summation of individual SWFRs. For the 24-whiskers array (Figure 7E), the magnitude values obtained *in vivo* were 11.3–16.8x weaker (mean ± SD = 13.7 ± 1.1), with the location of strongest activity not more or less overestimated by the model compared to elsewhere in barrel cortex. Similarly for the 4-whiskers (Figure 7F), the magnitude values obtained *in vivo* was also overestimated by modeling, although differences in magnitude ranged between 2.0–3.6x (mean ± SD = 2.6 ± 0.4) and therefore were not as striking as for the 24-whiskers array but still occurred relatively uniformly across barrel cortex.

## Discussion

In the present study, both *in vivo* and modeling approaches are used to characterize the rat barrel cortex response to stimulating more than one whisker (MWFR). We find that the MWFR of 24 whiskers possesses only a single peak that is located centrally within a large spread of progressively decaying activity, ultimately resembling a relatively symmetric activity mountain with a single central peak (Figures 2, 3). By explicitly incorporating SWFRs, which are large in spread and thus highly overlapping, and their interaction with each other (linear summation) into a simple model of MWFRs, we are able to predict salient properties of the 24-whiskers MWFR including the single central peak obtained *in vivo* (Figure 4). Furthermore, direct manipulation of cortical activation spreads by locally injecting lidocaine into the cortex distal to the MWFR peak activity leads to results in support of MWFR dependence on SWFR interactions (Figure 5). Findings are extended to a different combination of whiskers (subgroup of four neighboring whiskers within the 24-whiskers array; Figure 6). Last, we find that *in vivo*-MWFRs exhibit no or relatively modest increase in response magnitude and area but interestingly a reduction in variability of these response properties compared to an SWFR (Figure 7).

The finding of the 24-whiskers' MWFR resembling a relatively symmetrical activity mountain with only a single central peak (Figure 3) indicates that the cortical response to stimulating many whiskers transcends one-to-one topography to culminate in a single peaked activation spread that no longer conveys information about individual identities of the stimulated whiskers. Our modeling (Figure 4) and pharmacological (Figure 5) results shed some insight into the mechanism underlying this single peaked cortical response. Once interactions between SWFRs are taken into consideration (Figures 4A–C), the single central peak as well as other properties of the 24 whiskers' MWFR obtained *in vivo* (Figures 4D–F) can be successfully predicted. Furthermore, the dependence of MWFRs on highly overlapping and hence interacting SWFRs is directly confirmed *in vivo* (Figure 5). Our combined modeling and pharmacological findings indicate SWFRs and their interactions play a role in defining salient properties of the cortical response to stimulation of many whiskers. Our pharmacological results (Figure 5) also establish that SWFR interactions responsible for single-peaked MWFRs occur at the cortical level (as opposed to single-peaked MWFRs already established subcortically and passively transmitted to the cortex), which is in line with other evidence. Already, it has been demonstrated that the SWFR's large spread of activity occurs intracortically based on cortical transection experiments and anatomical tracer experiments implicating an underlying large spread of long-range intracortical horizontal projections (Frostig et al., 2008). Hence, anatomical infrastructure is in place to support SWFR interactions at the cortical level. Also, the response of cortical neurons have been found to differentiate between stimulation of a single (principal) whisker vs. a group of whiskers comprising the principal whisker plus its adjacent whiskers whereas thalamic neurons do not (Hirata and Castro-Alamancos, 2008). Plus, peripheral somatosensory neurons exhibit equivalent response patterns for single point vs. multi-point skin stimulation (Gardner and Spencer, 1972a), suggesting minimal interactions between sensory neurons at the peripheral level.

While successful in predicting many MWFR properties, our modeling predictions greatly overestimate the absolute response magnitudes obtained *in vivo* (Figures 7E,F). The much lower response magnitudes observed *in vivo* are not artifactual given larger magnitudes are possible (see individual peak magnitude values for single whisker stimulation in Figure 7A). The overestimation by the model indicates the cortical response to stimulating many whiskers is dependent on SWFR summation interactions that are specifically sublinear in nature. Indeed, only by normalizing the model with a constant divisive term can we better visualize how well the model fits to the *in vivo* data (Figures 4F and 6H). Sublinear summation of SWFRs suggests that at least some interactions between SWFRs must be inhibitory. The normalization of the model using a constant divisive term may even be considered a rudimentary means to model inhibition of activity. Our imaging and electrophysiology (Figures 3, 6, 7) findings of no or modest increases in peak magnitude and area compared to single whisker stimulation also support inhibition of activity. The combined imaging, electrophysiology, and modeling findings would be in line with previous single unit findings on the barrel cortex response to two or few whiskers in support of activity inhibition (for pioneering work see Simons, 1983; Land and Simons, 1985; for review see Fox, 2008), as well as findings obtained at the population level using optical imaging of intrinsic signals (Goldreich et al., 1998) or voltage-sensitive dyes (Kleinfeld and Delaney, 1996; Civillico and Contreras, 2006). Limited studies have been conducted that specifically investigate the simultaneous stimulation of whiskers (Ghazanfar and Nicolelis, 1997; Shimegi et al., 1999; Mirabella et al., 2001; Hirata and Castro-Alamancos, 2008), as is the case in the present study. Our results based on both wide field-of-view imaging of total population response and electrophysiology recordings of neurons (Figures 3, 6, 7) agree with Mirabella et al. findings of increasing inhibition in cortical activity with increasing number of simultaneously stimulated whiskers.

As the whiskers of awake and behaving rats can be stimulated sequentially as well as simultaneously during active exploration, it would be relevant to determine how our present findings extend to sequential stimulation of the entire whisker array. Based on mapping field potentials (Benison et al., 2006) or single electrode recordings of neuronal responses (Drew and Feldman, 2007) in rat barrel cortex, it already has been shown that the spatial distribution of response properties such as latency (Benison et al., 2006) and peak magnitude (Benison et al., 2006; Drew and Feldman, 2007) can change in a topographical manner depending on the particular parameters of sequential whisker array stimulation. Future imaging studies can be conducted to determine whether sequential stimulation of the entire whisker array can still lead to a large cortical activation spread with a single peak, and if so, whether the location of peak activity can differ in a topographical manner according to sequential stimulation parameters. Also of relevance for future imaging investigation are the possible effects of other stimulation parameters such as frequency (5 Hz used in the present study). We have already shown that the peak magnitude and tangential spread of activity evoked by single whisker stimulation remains the same whether the whisker is deflected 5 times at a rate of 5 Hz (as in the present study) vs. deflected only once (Polley et al., 1999). Given the MWFR dependence on sublinear summation interactions of SWFRs described in the present study, it would be interesting to see whether this constancy in the SWFR despite changes in stimulation parameters lends itself to our MWFR results holding up for at least some stimulation parameters other than those investigated here.

Interestingly, the peak magnitude and area of the single peaked cortical response obtained in the present study showed marked decrease in variability (Figure 7). Interactions between SWFRs occurring at the cortical level provide the opportunity to pool activity from a large population of neurons that could contribute to the improved reliability in cortical response properties to stimulating many whiskers as reported here. Work by Celikel and Sakmann (2007) may point toward a behavioral relevance for such a purpose for SWFR interactions. While able to use a single whisker just as well as the entire whisker array to learn a whisker-dependent gap-crossing task, mice with intact whisker arrays require less time to gather necessary tactile information before successfully crossing the gap. It would be interesting to see whether this faster behavioral response time associated with the use of many whiskers is due to the whiskers initiating interactions between large SWFRs that in turn enable more reliable response properties.

Although no longer conveying one-to-one topographical information about stimulated whiskers, a barrel cortex response to many whiskers possessing a single central peak (Figure 3) would be in line with the concept of sensory funneling derived from seminal human psychophysical work by Georg von Békésy. He demonstrated that simultaneous stimulation of several separate and discrete skin sites (i.e., point stimuli) results in perception of a single stimulation site located centrally to the actual stimulation sites (Bekesy, 1967). Important follow-up research by Gardner and colleagues (Gardner and Spencer, 1972a,b; Gardner and Costanzo, 1980a,b; Gardner and Tast, 1981) extended von Békésy's findings by demonstrating that cortical activity itself can exhibit funneling properties (single, central peak of activity) and is predictive of perceptual funneling. Some of these findings have more recently been replicated using evoked potentials (Hashimoto et al., 1999) and functional imaging (Chen et al., 2003). Here, we extend these findings by showing that a single, central location of peak cortical activity resulting from SWFR interactions can occur in response to stimulating many instead of just a few sites. Interactions between large cortical activation spreads in general could serve as an underlying mechanism of previous funneling reports of cortical activity and their perception. If so, it would be interesting to see whether the awake and behaving rat perceives the stimulation of the 24 whiskers as some integrated perception of a single “broad” whisker located centrally within the array of 24-whiskers rather than a collection of individual stimulated whiskers. Such research pursuits should find useful the findings that when whisker stimulation is delivered in a repetitive manner (e.g., five whisker deflections delivered at 5 Hz rate) just one stimulus occurrence is sufficient for activity to peak at a single central site, even when as little as 4-whiskers are being stimulated (Figures 3D,E and 6C,D).

Last, we offer for consideration a more general implication for the functional organization of rat barrel cortex. The existence of SWFRs have already been repeatedly demonstrated with a variety of techniques including intrinsic signal optical imaging, voltage sensitive dye imaging, and traditional electrophysiology techniques (for reviews see Frostig, 2006 and Fox, 2008; for spread observed specifically beyond barrel cortex see Brett-Green et al., 2001; Ferezou et al., 2006, 2007; Frostig et al., 2008; Lim et al., 2012). Furthermore, SWFRs occur for a variety of whiskers and are supported by an existing network of horizontal intracortical projections (Brett-Green et al., 2001; Frostig et al., 2008). With respect to the present findings, the barrel cortex response to stimulating many whiskers has been found dependent on these large SWFRs and their interaction with one another. We posit that large SWFRs and their interaction may even provide an underlying neurophysiological mechanism for previous reports of perceptual and cortical activity funneling. Taken together, our study combined with accumulating evidence support the assertion that large SWFRs (Figures 4A,B) be considered alongside topography (Figure 1B) and cortical columns (Figure 1C) as a fundamental principle of barrel cortex organization. Interestingly, SWFRs are but one example of large cortical activation spreads evoked by spatially restricted stimulation (e.g., whisker occupies a point on the skin). Large activation spreads evoked by point stimulation appear ubiquitous, having been observed across various sensory modalities and animal species (Grinvald et al., 1994; Bakin et al., 1996; Brett-Green et al., 2001; Ferezou et al., 2006, 2007; Sharon et al., 2007; Frostig et al., 2008; Lim et al., 2012). Future research can be pursued to determine whether large cortical activation spreads following point stimulation can be deemed a fundamental principle of functional organization for not just rat barrel cortex but for the cortex in general.

### Conflict of interest statement

The authors declare that the research was conducted in the absence of any commercial or financial relationships that could be construed as a potential conflict of interest.
